# Preclinical Studies of Stem Cell Transplantation in Intracerebral Hemorrhage: a Systemic Review and Meta-Analysis

**DOI:** 10.1007/s12035-015-9441-6

**Published:** 2015-09-26

**Authors:** Yang Hu, Na Liu, Ping Zhang, Chao Pan, Youping Zhang, Yingxin Tang, Hong Deng, Miribanu Aimaiti, Ye Zhang, Houguang Zhou, Guofeng Wu, Zhouping Tang

**Affiliations:** 1 Department of Neurology, Tongji Hospital, Tongji Medical College, Huazhong University of Science and Technology, Wuhan, 430030 People’s Republic of China; 2 Department of Geriatrics Neurology, Huashan Hospital, Fudan University, Shanghai, 200040 People’s Republic of China; 3 Department of Neurology, Affiliated Hospital of Guizhou Medical University, Guizhou, 550004 People’s Republic of China

**Keywords:** Intracerebral hemorrhage, Stem cells, Meta-analysis, Clinical translation

## Abstract

To comprehensively evaluate the therapeutic effects on both functional and structural outcomes, we performed a meta-analysis of preclinical data on stem cell therapy in intracranial hemorrhage, thus providing optimal evidence and instruction for clinical translation. We searched online databases to identify eligible studies based on unmodified stem cell transplantation in intracranial hemorrhage (ICH). From each study, we extracted data regarding neurobehavioral and histological outcomes in order to analyze the comprehensive effective sizes according to the most important clinical parameters (seven indices) and to explore any potential correlation through meta-regression. We analyzed 40 eligible studies including 1021 animals and found a significant improvement in both behavioral and structural outcomes with the median effect size of 1.77 for modified Neurological Severity Score, 1.16 for the modified placement test, 1.82 for the rotarod test, and 1.24 for tissue loss reduction. The meta-regression results revealed that intracerebral administration was the most effective for behavioral and structural recovery post-ICH; mesenchymal stem cells shared comparable therapeutic effects with neural stem cells. Delayed therapy, applied more than 1 week after ICH, showed the greatest improvement of structural outcomes. Stem cell therapy showed significant improvement on behavioral and structural outcomes of ICH animals with relatively large effect sizes. However, the practical efficacy of the therapy is likely to be lower considering poor study quality and non-negligible publication bias. Further, future research should interpret animal results cautiously considering the limited internal and external validity when referring to the design of both animal studies and clinical trials.

**Electronic supplementary material:**

The online version of this article (doi:10.1007/s12035-015-9441-6) contains supplementary material, which is available to authorized users.

## Introduction

Intracerebral hemorrhage (ICH), which results from rupture of blood vessels in the brain, remains a public health concern because it leads to high rates of mortality and disability in adults [1]. Hematoma expansion and brain edema are two important complications that might occur during the acute and subacute phases of ICH; both are known to exacerbate brain injury [2, 3]. Management of ICH is largely carried out via mechanical removal of the hematoma, pharmacological prevention of edema formation, and reduction in intracranial pressure. The intent of all of these methods is to limit further brain injury and associated complications. Unfortunately, compared with ischemic stroke, ICH has received less research attention, and, to date, no currently available medical therapy has shown a consistent or unambiguous benefit in functional outcomes after ICH [4, 5].

Stem cells—including embryonic stem cells (ESCs), somatic stem cells, and induced pluripotent stem cells (iPSCs)—are characterized by their capacity for self-renewal and multiple differentiation. Mesenchymal stem cells (MSCs) have long been used in preclinical and clinical research [6, 7], and stem cell therapy has become one of the most promising strategies for the treatment of comprehensive human diseases, such as ischemic heart disease, autoimmune diseases, and neurological disorders [8–11]. The general intent of stem cell therapy is to replace lost cells or restore the function of damaged tissues by introducing with the capacity of secreting multiple growth factors, cytokines, and neurotrophins. However, the underlying mechanisms are far more complex and still not fully understood at present [12–14].

A number of reports on stem cell transplantation in ICH animal models indicate improved neurobehaviors or attenuation of the hematoma [15–17]. Among various stem cells, MSCs and neural stem cells (NSCs) are the most widely used and offer great promise in ICH repair. However, the administration dose, route, time interval, cell source, type, manipulation, and quality score in each study are so divergent that the overall therapeutic effect is difficult to evaluate. Consequently, the optimal pattern of cell therapy remains unclear [18]. To clarify the current situation and future research directions in cell therapy as a treatment for ICH, we collected data from all relevant studies and performed a meta-analysis to quantify both the functional and structural efficacy of stem cell therapy. Among these studies, the most widely used empirical tests to assess behavioral outcome of ICH include the modified neurological severity score (mNSS), modified limb placement test (mLPT), and rotarod test [19, 20]. The studies included in the meta-analysis are graded strictly according to Collaborative Approach to Meta-Analysis and Review of Animal Data from Experimental Stroke (CAMARADES) checklists [21].

## Methods

### Search Strategy

Studies of stem cell-based therapy for ICH animal models were retrieved from PubMed and Medline through April 2015 using the following search terms: (progenitor OR stem OR bone marrow OR mesenchymal OR haematopoietic) AND (basal ganglion hemorrhage OR brain hemorrhage OR brain ventricle hemorrhage OR cerebellum hemorrhage OR intracranial hemorrhage OR hemorrhage stroke). Two researchers worked independently on these searches (Yang Hu and Na Liu). Retrieved articles and abstracts, including secondary references, were thoroughly scanned and reviewed by the two researchers, either. Eligible studies were reviewed in duplicate to determine whether or not to be included in the meta-analysis.

### Inclusion and Exclusion Criteria

The meta-analysis included controlled studies that compared neurobehavioral outcomes—with or without structural outcomes—in wild-type (nontransgenic) animal groups. In these studies, ICH was induced using autologous blood or collagenase injection, after which subjects were treated with allogeneic or autologous stem cells and a placebo (saline, culture medium, or similar vehicle). The meta-analysis excluded studies that used nontraumatic models of hemmorrhagic injury or individual comparisons that were not reported or from which we could not calculate the number of animals, the mean outcome, or the variance in each group. We also excluded studies that used substantially manipulated stem cells, including cells differentiated into mature neural cells, co-transplanted with other stem cells, or transfected with genes other than labeling or tracing markers.

### Data Collection

The following items from the eligible studies were independently extracted by the two researchers: general study information (first author, publication date), ICH model, cell characteristics (cell type, source, dose, delivery route), recipient animal species, functional outcome (neurobehavioral score measured on any scale, modified limb placement test, rotarod test), structural outcome (brain water content or tissue loss reduction), and study quality index.

From each experiment, the researchers extracted, without exception, all available data from reported outcomes available, text, and graphs. When only graphic presentations were available, values for mean and standard deviation (SD) were obtained via calibrating images using GetData Graph Digitizer software. If the study included more than one experimental group differentiated by delivery time or cell number that was compared against a common control group, these parallel groups would be included separately as independent experiments and the control group size divided equally among the numbers of treatment groups. If the SD was not directly reported, it was calculated by multiplying the reported standard error (SE) by the square root of the group size. Where functional outcome was measured at different times, only the most recent one was extracted.

### Methodological Quality of Studies

The quality of each experiment was assessed according to the CAMARADES checklists, which consist of the following: (1) publication in a peer-reviewed journal, (2) control of animals’ temperature, (3) randomized treatment allocation, (4) treatment allocation concealment, (5) blind assessment of outcome, (6) avoidance of anesthetics with known marked intrinsic neuroprotective activity such as ketamine, (7) reporting of a sample size calculation, (8) statement of compliance with regulatory requirements, (9) statement of potential conflicts of interest, and (10) use of animals with relevant comorbidities.

### Statistical Analyses

Effect size was calculated to be the absolute difference with 95 % confidence interval (CI) between stem cell treated animals and comparable controls. For the rotarod test, in contrast to the other three measures, outcome values were multiplied by −1 because the value was positively correlated with its outcome. The DerSimonian-Laird random meta-analysis model and Hedges calculation were adopted to determine a comprehensive estimation of effect size with standard mean differences, and meta-analysis was performed using Stata software (version 12.1). Generally, an effect size of 0.2 was defined as a small effect, 0.5 as medium and 0.8 as large. A probability value of *P* < 0.05 was considered statistically significant. Seven intriguing clinical parameters were used to stratify the effect size: cell source (autologous, allogeneic, or xenogeneic); cell type (NSCs, MSCs, or other stem cells); cell dose (<1E6, 1E6–5E6, >5E6); delivery time (0–8 h, 24 h, 1–7 days, or >7 days); randomization; blind review by operator; and total quality score. Meta-regression and pre-specified subgroup analysis were used to explore the potential relationships between mNSS and tissue loss and the aforementioned parameters. Publication bias was examined using funnel plots, and significant publication bias was assessed using Egger regression. If necessary, any non-negligible bias would be corrected using the Duval and Tweedie trim-and-fill approach.

## Results

### Study Characteristics

**Fig. 1:**
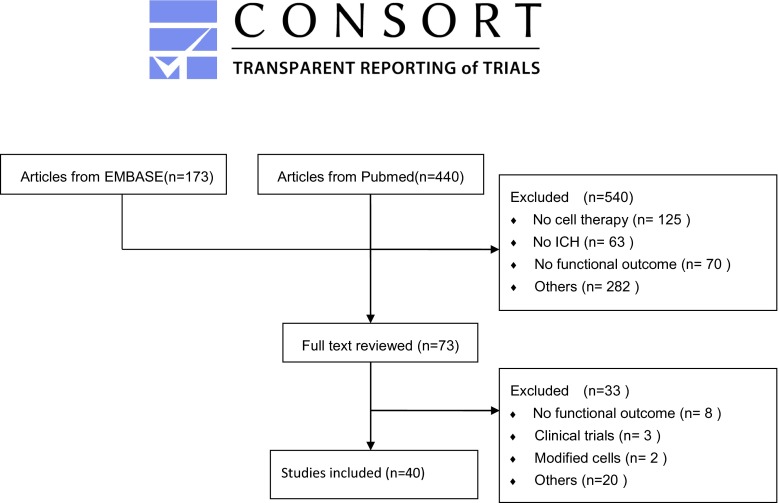
Flow diagram of preferred reporting items for systematic reviews and meta-analyses (PRISMA) (created by Microsoft Word)

Electronic searching identified 440 articles in PubMed and 173 articles in EMBASE. From among these, 40 eligible studies containing 589 cell treatment animals and 432 comparable controls were eventually included in our meta-analysis (Fig. 1). Twenty of these studies demonstrated improvement in mNSS, and seven of the 11 studies reported decreased tissue loss. The vast majority of the studies (62.5 %) used MSCs derived from bone marrow, adipose tissue, or umbilical blood, and, except for two studies that used iPSCs, the remaining studies used NSCs. In 33 of the 40 studies, a collagenase-induced ICH model was adopted, while the remaining studies adopted an autologous blood-induced ICH model.

### Quality Score

The quality scores varied from 1 to 7, with a mean value of 4.45. According to our statistical results, all the articles were published in a peer-reviewed journal; 37.5 % described control of temperature, 75 % declared randomization to treatment group, 60 % stressed blind assessment of outcome, 50 % avoided usage of ketamine as anesthetics, 90 % claimed compliance with animal welfare regulations, and 30 % stated a conflict of interest (Table 1; SI File).

**Table 1:** Percentage of included studies satisfying each criterion of CAMARADES checklists

Quality score criterion	Percentage of qualified studies (%)
Published in peer-reviewed journal	100
Control of temperature	37.5
Randomization to treatment group	75
Allocation concealment	2.5
Blinded assessment of outcome	60
Avoidance neuroprotective anesthetics	50
Sample size calculation	0
Compliance with animal welfare regulations	90
Statement of conflict of interest	30
Use of animals with relevant comorbidities	0

### Meta-analysis

**Fig. 2:**
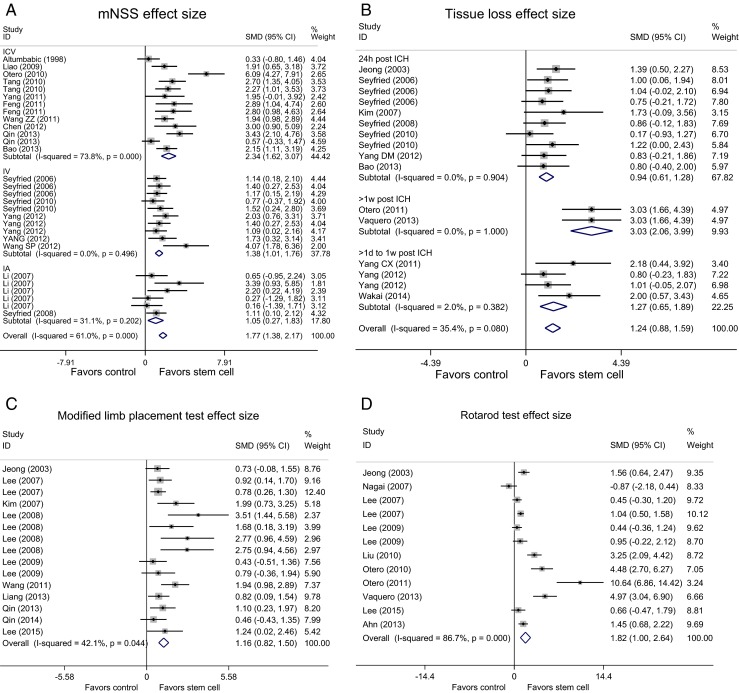
Forest plot shows mean effect size and 95 %CI for **a** mNSS, **b** tissue loss reduction, **c** MLPT, and **d** RR. *mNSS* modified neurological severity score, *MLPT* modified limb placement test, *RR* rotarod test, *ICV* intracerebral, *IV* intravenous, *IA* intra-artery (created by stata)

Overall, use of stem cell therapy showed an improvement in both functional and structural outcomes post-ICH, and all four effect sizes were statistically significant (1.77 for mNSS, 1.16 for MLPT, 1.82 for RR, and 1.24 for tissue loss reduction) (Fig. 2). Observed heterogeneity was higher than what would be expected from sampling error alone and could not be ignored (*τ*^2^ = 0.6777, *I*^2^ = 61 % for mNSS; *τ*^2^ = 0.1751, *I*^2^ = 42.1 % for MLPT; *τ*^2^ = 1.6451, *I*^2^ = 86.7 % for RR; *τ*^2^ = 0.1833, *I*^2^ = 35.4 % for tissue loss reduction). For the two outcomes with the largest amount of published data—mNSS and tissue loss reduction—meta-regression was used to explore potential contributions to heterogeneity of the parameters mentioned above.

### Meta-regression for Functional and Structural Outcomes

**Fig. 3:**
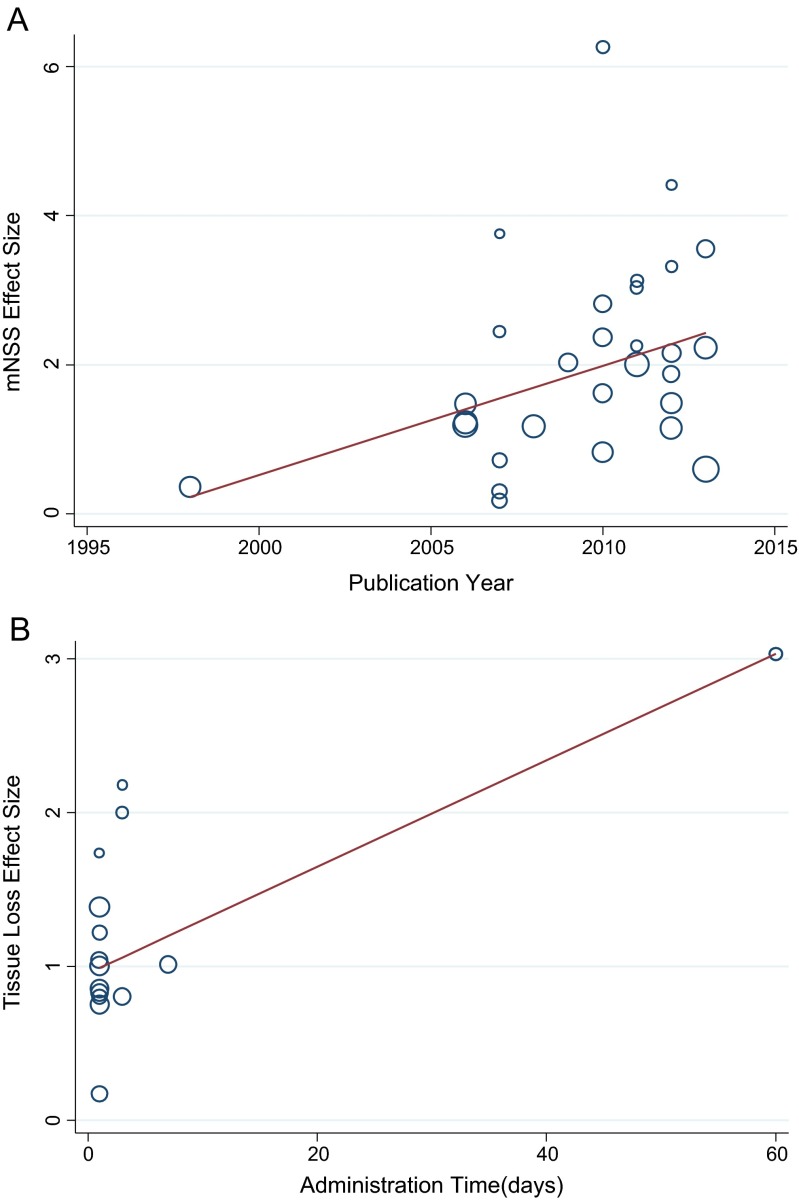
Meta-regression results for **a** effect size of mNSS positively correlated with publication year and **b** effect size of tissue loss reduction positively correlated with administration time (created by stata)

For mNSS, publication year remained the only significant predictor (*P* = 0.036); the more recently the research was conducted, the larger the effect sizes were (Fig. 3a). For tissue loss reduction, administration route and time remained as significant predictors (*P* = 0.005 for administration route; *P* = 0.002 for administration time); in fact, intracerebral (ICV) injection was demonstrated to be the most effective route for improvement of both mNSS and tissue loss reduction, more so than intravenous (IV) and intra-arterial (IA) injections. In tissue loss reduction, cell therapy initiated more than 1 week post-ICH showed the greatest difference, followed next by cell therapy initiated within 1 week post-ICH, and then therapy initiated with 24 h (mean effect size 3.027 vs 1.270 vs 0.942; *P* = 0.002; Fig. 3b). None of the other clinical parameters were predictors for effect size, nor were any of the three quality parameters (total quality score, randomization, and allocation concealment). Additionally, subgroup analysis revealed that MSCs shared a comparable effect size with NSCs, as did allogenic cells with xenogenic cells (Tables 2 and 3).

**Table 2:** Results of mNSS from subgroup analysis of parameters

Parameters					
Dose	<1E6	1E6–5E6	>5E6		
	1.773 (1.134, 2.411)	1.954 (1.378, 2.530)	1.273 (0.516, 2.029)		NS
Route	ICV	IV	IA		
	2.344 (1.617, 3.071)	1.384 (1.010, 1.757)	1.049 (0.270, 1.829)		*P* = 0.036
Time	0–8 h	24 h	>1 day–1 week	>1 week	
	4.071 (1.784, 6.358)	1.500 (1.086, 1.915)	2.373 (1.652, 3.094)	0.543 (−0.215, 1.300)	NS
Immunity	Allogenic	Xenogenic			
	1.950 (1.210, 2.690)	1.617 (1.217, 2.017)			NS
Type	NSC	MSC	IPS		
	1.459 (0.728, 2.190)	1.896 (1.418, 2.374)	1.960 (−0.843, 4.763)		NS
Blinding	Nonblinded	Blinded			
	1.655 (1.047, 2.264)	1.890 (1.368, 2.413)			NS
Randomization	Nonrandomized	Randomized			
	1.926 (0.861, 2.991)	1.769 (1.349, 2.189)			NS

Effect size of mNSS from subgroup analysis of clinical parameters presented as mean with 95 % confidence interval

*ICV* intracerebral, *IV* intravenous, *IA* intra-artery, *NS* not significant

**Table 3:** Results of tissue loss reduction from subgroup analysis of parameters

Parameters				
Dose	<1E6	1E6–5E6	>5E6	
	1.147 (0.208, 2.087)	1.332 (0.907, 1.756)	0.753 (−0.212, 1.717)	NS
Route	ICV	IV	IA	
	2.174 (1.282, 3.065)	0.955 (0.621, 1.290)	0.856 (−0.120, 1.833)	*P* = 0.005
Time	24 h	>1 day–1 week	>1 week	
	0.942 (0.606, 1.278)	1.270 (0.648, 1.892)	3.027 (2.062, 3.993)	*P* = 0.002
Immunity	Allogenic	Xenogenic		
	1.882 (0.906, 2.859)	0.984 (0.654, 1.315)		NS
Type	NSC	MSC		
	1.557 (0.803, 2.312)	1.189 (0.790, 1.588)		NS
Blinding	Nonblinded	Blinded		
	1.225 (0.712, 1.738)	1.269 (0.740, 1.797)		NS
Randomization	Nonrandomized	Randomized		
	1.137 (0.700, 1.573)	1.293 (0.718, 1.868)		NS
Location	Toatal	Striatum		
	2.143 (1.200, 3.085)	0.973 (0.659, 1.286)		*P* = 0.008

Effect size of tissue loss reduction from subgroup analysis of clinical parameters presented as mean with 95 % confidence interval

*ICV* intracerebral, *IV* intravenous, *IA* intra-artery, *NS* not significant

### Publication Bias

Visual inspection of funnel plots of mNSS and MLPT revealed asymmetry. This was consistent with the results from the Egger test, which suggested prominent publication bias to the left of the estimate (*P* = 0.004 for mNSS; *P* = 0.001 for MLPT). However, after correcting for the bias, the pooled analysis incorporating the hypothetical studies remained statistically significant, and all four calibrated effect sizes exceed 1 (1.367 for mNSS and 1.053 for MLPT).

## Discussion

This meta-analysis including 40 studies and 1021 animals suggests the following: (1) ICH animals benefited greatly from stem cell therapy, with treatment using both NSCs and MSCs exhibiting statistically significant improvement in functional and structural outcomes. (2) ICV proved to be the most effective administration route compared with IV or IA to improve mNSS and decrease tissue loss. (3) Allogenic and xenogenic cells exhibited similarly beneficial effect size for mNSS, although the former facilitated lesion site recovery to a larger extent. (4) Intervention time was positively correlated with effect size in tissue loss reduction but not in mNSS. (5) Stem cell-based therapy shows promise in treating ICH animals based on our study, but determining the efficacy is inevitably confounded by poor study quality and publication bias. Therefore, our conclusions should be tested in more rigorously designed studies and carefully interpreted in relation to the design of future clinical translation or animal studies.

The categories of stem cells already used for ICH animals are NSCs [16, 17], ESCs [22], human MSCs [23], human bone marrow stromal cells (HBMSCs) [24], adipose-derived stem cells [25], human umbilical cord blood cells (HUCBCs), and human iPSCs [26]. Our meta-analysis included all but ESCs, with the majority being MSCs derived from various tissues. Our pre-specified subgroup analysis stratified by cell type showed that MSCs had a greater positive impact on mNSS, while NSCs were more effective in decreasing tissue loss. Although post hoc meta-regression detected no correlation between cell type and effect size, this distinction between the two cells types might be explained by their different therapeutic mechanisms. To be specific, because of the unique capacity of NSCs to differentiate into functional neural cells, reduced tissue loss might result from donor NSCs replacing damaged neurons, although very few studies have rigorously examined the electrophysiological and transmitter synthesis function of transplanted NSCs in vivo [27, 28]. But again, this is likely the result of NSC secretion of several cytokines after ICH, which, as recent studies have shown, attenuate systemic inflammatory response [10, 29, 30]. Even with these recent studies, this unique mechanism requires further exploration in future research work. Compared with NSCs, the effectiveness of MSCs as documented in models of neurological disease is attributed mainly to the cells’ ability to secrete various neurotrophins, cytokines, or growth factors, including brain-derived neurotrophic factor (BDNF), nerve growth factor (NGF), basic fibroblast growth factor (bFGF), and vascular endothelial growth factor (VEGF), all of which possibly contribute to the functional recovery of the subventricular zone (SVZ) in the adult central nervous system [23, 31, 32]. The number of transplanted MSCs that express physical and biochemical characteristics of neural cells is extremely low, so virtually none of them are able to be integrated into functional neural circuitry [33]. Nevertheless, the underlying mechanisms are far more complex and still not fully understood at present. Given the current state of research, we find that MSCs share comparable therapeutic effects with NSCs in ICH, which challenges the obsolete view that NSCs might be considered the most appropriate cells for treating nervous system diseases like ICH. Therefore, MSCs seem to be a promising cell source for future clinical application because of their similarity to NSCs in their effects, and yet they require a less invasive and delicate isolation procedures than NSCs [34].

Of additional concern are the immunological issues commonly associated with the process of allogenic or xenogenic cell transplantation. Once exogenous cells trigger an intrinsic immune rejection in vivo, the transplanted cells will be eliminated by the activated immune system, a process that not only destroys the cell functions, but also causes damage to the hosts [35, 36]. Considering that majority of cells included in our analysis were MSCs, which can exert overt regulation on the host’s immune system, no obvious immune rejections were reported, even in the xenogenic cell transplantation group not simultaneously treated with immunosuppressants. In our subgroup analysis, the difference in effect size between allogenic and xenogenic cells was not statistically significant (*P* > 0.05).

Administration route and dose are typically the focus if cell therapy is applied in clinical situations. In our analysis, the intracerebral route seems to have a distinct advantage over both the IV and intracarotid routes. Compared with ICV, either IV or IA is relatively less invasive and more convenient to manipulate. Temporarily disregarding the inconvenience of ICV, it does show superiorities over IV route in that it can rapidly and directly target the lesion site while avoiding spleen phagocytosis and retardation of brain blood barrier retention [37–39]. More importantly, it is possible to combine cell therapy administered through ICV with hematoma evacuation after ICH [40, 41]. At the same time, cell viability might decrease significantly once the cells are exposed directly via ICV to a heavily inflammatory local environment. Therefore, exploring innovative and effective alternative delivery routes such as intranasal delivery might be a useful future research direction. The majority of the included studies tended to initiate the cell therapy at 24 h post-ICH, followed by therapy initiation within 1 week post-ICH; only four exceeded more than 1 month before beginning treatment, and two of those did not begin treatment until 2 months post-ICH. We found a positive correlation between intervention time and structural effect size, an observation that might be explained by the fact that a substantial number of studies conducted a final structural assessment before reaching the plateau recovery phase. However, it is important to keep in mind that subgroup analyses can only generate hypotheses rather than confirming them.

Assessment of the methodological quality of animal studies should follow strict criteria regarding clinical trials because of the potential that the design of these trials might influence their results [21]. The average quality score according to the CAMARADES list was lower than we had expected. The percentage of descriptive randomization and blindness were 75 and 60, respectively, and none of the studies explicitly stated the specific procedures followed in conducting the experiments. Additionally, half of the studies adopted ketamine as the anesthetic regardless of its well-known neuroprotective effects. Intriguingly, randomization, blinding, and total quality score were not significant predictors for effect size, which can be partially attributed to the ambiguous description of the methods. As a result, we could not predict the effect size for subsequent studies based on quality scores. To reduce the risk of bias and given this evidence of the poor reporting of measures, we encourage future research to report both detailed methodology and measures performance in the field. Both Funnel plots and Egger tests detected obvious publication bias, so a trim-and-fill approach was adopted to correct the bias, although the modified effect sizes were still remarkable. As might be expected, studies reporting more positive outcomes are more likely to be accepted for publication, especially in animal studies.

There is significant work to be done when it comes to clinical translation [42]. First of all, the study subjects are primarily rats, which share limited similarities with human beings in anatomical and biochemical properties, unlike porcines or primates, which can mimic human pathological process more vividly and precisely [43, 44]. Thus, because the neurobehavioral outcomes for rats cannot be directly extended to human beings, more reliable results could be obtained by using multiple species. Secondly, although ICH is generally the result of ruptured vessels affected by hypertension-related degenerative changes or the natural course of cerebral amyloid angiopathy, current study models are almost entirely based on healthy animals without any chronic comorbidities, letting alone hypertension [45]. Furthermore, the majority of ICH patients are elderly, while study animals are selected from a fixed age group with a high proportion of youngsters. In terms of the meta-analysis itself, the internal and external validity are obviously confounded by poor study quality and non-negligible publication bias. Consequently, the efficacy of stem cell therapy for ICH will be amplified to some extent because studies that remain more often contain neutral or negative data.

Although several reviews and meta-analyses focusing on cell therapy for neurological diseases have been published, this is the first meta-analysis to concentrate specifically on stem cells used for ICH in animal models [46, 47]. Although the obvious effect size for ischemic stroke of MSCs alone or for all stem cells has been emphasized, the pathophysiological features of ischemic and hemorrhagic stroke diverge in many aspects, so great caution should be taken when comparing findings for each disease [48].

According to our study, stem cell-based therapies may offer promise in treating ICH, and, the therapeutic effect of stem cells in this application seems obvious based on preclinical research. However, future research should interpret animal results cautiously considering the limited internal and external validity when referring to the design of both animal studies and clinical trials.

## Electronic Supplementary Material

Below is the link to the electronic supplementary material.

ESM 1 (PDF 149 kb)

## Glossary

CAMARADES: Collaborative Approach to Meta-Analysis and Review of Animal Data from Experimental Stroke
ICH: Intracerebral hemorrhage
IPS: Induced pluripotent stem cell
MLPT: Modified limb placement test
mNSS: Modified neurological severity score
MSC: Mesenchymal stem cell
NSC: Neural stem cell
RR: Rotarod test
