# Supplementary material for: Preclinical Studies of Stem Cell Transplantation in Intracerebral Hemorrhage: a Systemic Review and Meta-Analysis
Source: Mol Neurobiol. 2015 Sep 26;53(8):5269–77. doi: 10.1007/s12035-015-9441-6 (PMC5012148; doi:10.1007/s12035-015-9441-6)
Supplement: Supplementary file 1 — (PDF 149 kb) [file 12035_2015_9441_MOESM1_ESM.pdf]

Preclinical Studies of Stem Cell Transplantation in Intracerebral Hemorrhage:

A Systemic Review and Meta-Analysis

Author Information

Yang Hu[1], Na Liu[1], Ping Zhang[1], Chao Pan[1], Youping Zhang[1], Yingxin Tang[1], Hong Deng[1], Miribanu Aimaiti[1], Ye Zhang[1], Houguang Zhou[2], Guofeng Wu[3] and Zhouping Tang[1]

Authors institutional affiliations:

[1]Department of Neurology, Tongji Hospital, Tongji Medical College, Huazhong University of Science and Technology, Wuhan 430030, P. R. China;

[2]Department of Geriatrics Neurology, Huashan Hospital, Fudan University, Shanghai, 200040, P.R. China;

[3]Department of Neurology, Affiliated Hospital of Guizhou Medical University, Guizhou, 550004, P. R. China;

Corresponding author:

Dr. Zhouping Tang Tel: 0086-27-83663337; Fax: 0086-27-83663337; Email: ddjtzp@163.com

Publication	year		(1)	(2)	(3)	(4)	(5)	(6)	(7)	(8)	(9)	(10)	total
Altumbabic	1998	[1]	+		+		+	+		+			5
Jeong S	2003	[2]	+	+	+		+			+			5
Nan	2005	[3]	+		+					+	+		4
Seyfried	2006	[4]	+										1
Zhang	2006	[5]	+		+								2
Kim	2007	[6]	+				+			+			3
Lee	2007	[7]	+	+	+		+			+	+		6
Lee	2007	[8]	+	+			+			+			4
Li	2007	[9]	+		+			+		+			4
Nagai	2007	[10]	+	+									2
Lee	2008	[11]	+	+	+		+			+			5
Seyfried	2008	[12]	+							+			2
Fatar	2008	[13]	+		+			+		+			4
Liao	2009	[14]	+	+			+	+		+	+		6
Lee	2009	[15]	+	+	+		+			+			5
Lee	2009	[16]	+	+	+		+			+			5
Lee	2010	[17]	+	+			+			+			4
Liu	2010	[18]	+	+	+					+	+		5
Otero	2010	[19]	+		+			+		+			4
Tang	2010	[20]	+		+			+		+			4
Seyfried	2010	[21]	+	+	+					+			4
Yang	2011	[22]	+					+		+			3
Feng	2011	[23]	+		+	+	+			+			5
Otero	2011	[24]	+		+			+		+			4
Wang	2011	[25]	+		+		+	+		+	+		6
Yang	2012	[26]	+		+		+	+		+			5
Chen	2012	[27]	+		+		+	+		+	+		6
Yang	2012	[28]	+	+	+		+	+		+			6

Ahn	2012	[29]	+		+		+	+		+	+		6
Wang	2012	[30]	+		+		+	+		+			5
Chen	2012	[31]	+		+					+			3
Seghatoleslam	2012	[32]	+		+		+			+			4
Vaquero	2013	[33]	+		+		+	+		+	+		6
Qin	2013	[34]	+		+		+			+	+		5
Qin	2013	[35]	+		+		+	+		+	+		6
Liang	2013	[36]	+				+	+		+			4
Bao	2013	[37]	+	+	+		+	+		+	+		7
Seghatoleslam	2013	[38]	+		+		+			+			4
Wakai	2014	[39]	+	+				+		+	+		5
Lee	2015	[40]	+	+	+			+					4
**total**			40	15	30	1	24	20	0	36	12	0	
**percentage**			100	37.5	75	2.5	60	50	0	90	30	0	

Publication quality check list:

(1) Publication in a peer reviewed journal
(2) Statement describing control of temperature
(3) Randomisation to treatment group
(4) Allocation concealment
(5) Blinded assessment of outcome
(6) Avoidance of anaesthetic with known marked intrinsic neuroprotective properties
(7) Sample size calculation
(8) Compliance with animal welfare regulations
(9) Statement of any potential conflict of interest
(10) Use of animals with relevant comorbidities (hypertension or diabetes)

1. Altumbabic M, Del Bigio MR (1998) Transplantation of fetal brain tissue into the site of intracerebral hemorrhage in rats. Neuroscience letters 257 (2):61-64
2. Jeong SW, Chu K, Jung KH, Kim SU, Kim M, Roh JK (2003) Human neural stem cell transplantation promotes functional recovery in rats with experimental intracerebral hemorrhage. Stroke; a journal of cerebral circulation 34 (9):2258-2263. doi:10.1161/01.str.0000083698.20199.1f
3. Nan Z, Grande A, Sanberg CD, Sanberg PR, Low WC (2005) Infusion of human umbilical cord blood ameliorates neurologic deficits in rats with hemorrhagic brain injury. Annals of the New York Academy of Sciences 1049:84-96. doi:10.1196/annals.1334.009

4. Seyfried D, Ding J, Han Y, Li Y, Chen J, Chopp M (2006) Effects of intravenous administration of human bone marrow stromal cells after intracerebral hemorrhage in rats. Journal of neurosurgery 104 (2):313-318. doi:10.3171/jns.2006.104.2.313
5. Zhang H, Huang Z, Xu Y, Zhang S (2006) Differentiation and neurological benefit of the mesenchymal stem cells transplanted into the rat brain following intracerebral hemorrhage. Neurological research 28 (1):104-112. doi:10.1179/016164106x91960
6. Kim JM, Lee ST, Chu K, Jung KH, Song EC, Kim SJ, Sinn DI, Kim JH, Park DK, Kang KM, Hyung Hong N, Park HK, Won CH, Kim KH, Kim M, Kun Lee S, Roh JK (2007) Systemic transplantation of human adipose stem cells attenuated cerebral inflammation and degeneration in a hemorrhagic stroke model. Brain research 1183:43-50. doi:10.1016/j.brainres.2007.09.005
7. Lee HJ, Kim KS, Kim EJ, Choi HB, Lee KH, Park IH, Ko Y, Jeong SW, Kim SU (2007) Brain transplantation of immortalized human neural stem cells promotes functional recovery in mouse intracerebral hemorrhage stroke model. Stem cells (Dayton, Ohio) 25 (5):1204-1212. doi:10.1634/stemcells.2006-0409
8. Lee HJ, Kim KS, Park IH, Kim SU (2007) Human neural stem cells over-expressing VEGF provide neuroprotection, angiogenesis and functional recovery in mouse stroke model. PloS one 2 (1):e156. doi:10.1371/journal.pone.0000156
9. Li F, Liu Y, Zhu S, Wang X, Yang H, Liu C, Zhang Y, Zhang Z (2007) Therapeutic time window and effect of intracarotid neural stem cells transplantation for intracerebral hemorrhage. Neuroreport 18 (10):1019-1023. doi:10.1097/WNR.0b013e328165d170
10. Nagai A, Kim WK, Lee HJ, Jeong HS, Kim KS, Hong SH, Park IH, Kim SU (2007) Multilineage potential of stable human mesenchymal stem cell line derived from fetal marrow. PloS one 2 (12):e1272. doi:10.1371/journal.pone.0001272
11. Lee ST, Chu K, Jung KH, Kim SJ, Kim DH, Kang KM, Hong NH, Kim JH, Ban JJ, Park HK, Kim SU, Park CG, Lee SK, Kim M, Roh JK (2008) Anti-inflammatory mechanism of intravascular neural stem cell transplantation in haemorrhagic stroke. Brain : a journal of neurology 131 (Pt 3):616-629. doi:10.1093/brain/awm306
12. Seyfried DM, Han Y, Yang D, Ding J, Savant-Bhonsale S, Shukairy MS, Chopp M (2008) Mannitol enhances delivery of marrow stromal cells to the brain after experimental intracerebral hemorrhage. Brain research 1224:12-19. doi:10.1016/j.brainres.2008.05.080
13. Fatar M, Stroick M, Griebe M, Marwedel I, Kern S, Bieback K, Giesel FL, Zechmann C, Kreisel S, Vollmar F, Alonso A, Back W, Meairs S, Hennerici MG (2008) Lipoaspirate-derived adult mesenchymal stem cells improve functional outcome during intracerebral hemorrhage by proliferation of endogenous progenitor cells stem cells in intracerebral hemorrhages. Neuroscience letters 443 (3):174-178. doi:10.1016/j.neulet.2008.07.077
14. Liao W, Zhong J, Yu J, Xie J, Liu Y, Du L, Yang S, Liu P, Xu J, Wang J, Han Z, Han ZC (2009) Therapeutic benefit of human umbilical cord derived mesenchymal stromal cells in intracerebral hemorrhage rat: implications of anti-inflammation and angiogenesis. Cellular physiology and biochemistry : international journal of experimental cellular physiology, biochemistry, and pharmacology 24 (3-4):307-316. doi:10.1159/000233255
15. Lee HJ, Kim MK, Kim HJ, Kim SU (2009) Human neural stem cells genetically modified to overexpress Akt1 provide neuroprotection and functional improvement in mouse stroke model. PloS one 4 (5):e5586. doi:10.1371/journal.pone.0005586
16. Lee HJ, Park IH, Kim HJ, Kim SU (2009) Human neural stem cells overexpressing glial cell line-derived neurotrophic factor in experimental cerebral hemorrhage. Gene therapy 16 (9):1066-1076. doi:10.1038/gt.2009.51

17. Lee HJ, Lim IJ, Lee MC, Kim SU (2010) Human neural stem cells genetically modified to overexpress brain-derived neurotrophic factor promote functional recovery and neuroprotection in a mouse stroke model. Journal of neuroscience research 88 (15):3282-3294. doi:10.1002/jnr.22474
18. Liu AM, Lu G, Tsang KS, Li G, Wu Y, Huang ZS, Ng HK, Kung HF, Poon WS (2010) Umbilical cord-derived mesenchymal stem cells with forced expression of hepatocyte growth factor enhance remyelination and functional recovery in a rat intracerebral hemorrhage model. Neurosurgery 67 (2):357-365; discussion 365-356. doi:10.1227/01.neu.0000371983.06278.b3
19. Otero L, Bonilla C, Aguayo C, Zurita M, Vaquero J (2010) Intralesional administration of allogeneic bone marrow stromal cells reduces functional deficits after intracerebral hemorrhage. Histology and histopathology 25 (4):453-461
20. Tang ZP, Xie XW, Shi YH, Liu N, Zhu SQ, Li ZW, Chen Y (2010) Combined transplantation of neural stem cells and olfactory ensheathing cells improves the motor function of rats with intracerebral hemorrhage. Biomedical and environmental sciences : BES 23 (1):62-67
21. Seyfried DM, Han Y, Yang D, Ding J, Shen LH, Savant-Bhonsale S, Chopp M (2010) Localization of bone marrow stromal cells to the injury site after intracerebral hemorrhage in rats. Journal of neurosurgery 112 (2):329-335. doi:10.3171/2009.2.jns08907
22. Yang C, Zhou L, Gao X, Chen B, Tu J, Sun H, Liu X, He J, Liu J, Yuan Q (2011) Neuroprotective effects of bone marrow stem cells overexpressing glial cell line-derived neurotrophic factor on rats with intracerebral hemorrhage and neurons exposed to hypoxia/reoxygenation. Lippincott Williams and Wilkins (351 West Camden Street, Baltimore MD 21201-2436, United States). http://ovidsp.ovid.com/ovidweb.cgi?T=JS&PAGE=reference&D=emed10&NEWS=N&AN=2011100967. Accessed (Yang) Department of Anatomy and Neurobiology, Tongji University School of Medicine, Shanghai, China 68
23. Feng M, Zhu H, Zhu Z, Wei J, Lu S, Li Q, Zhang N, Li G, Li F, Ma W, An Y, Zhao RC, Qin C, Wang R (2011) Serial 18F-FDG PET demonstrates benefit of human mesenchymal stem cells in treatment of intracerebral hematoma: a translational study in a primate model. Journal of nuclear medicine : official publication, Society of Nuclear Medicine 52 (1):90-97. doi:10.2967/jnumed.110.080325
24. Otero L, Zurita M, Bonilla C, Aguayo C, Vela A, Rico MA, Vaquero J (2011) Late transplantation of allogeneic bone marrow stromal cells improves neurologic deficits subsequent to intracerebral hemorrhage. Cytotherapy 13 (5):562-571. doi:10.3109/14653249.2010.544720
25. Wang Z, Cui C, Li Q, Zhou S, Fu J, Wang X, Zhuge Q (2011) Intracerebral transplantation of foetal neural stem cells improves brain dysfunction induced by intracerebral haemorrhage stroke in mice. Journal of cellular and molecular medicine 15 (12):2624-2633. doi:10.1111/j.1582-4934.2011.01259.x
26. Yang D, Han Y, Zhang J, Seyda A, Chopp M, Seyfried DM (2012) Therapeutic effect of human umbilical tissue-derived cell treatment in rats with experimental intracerebral hemorrhage. Brain research 1444:1-10. doi:10.1016/j.brainres.2012.01.024
27. Chen J, Tang YX, Liu YM, Chen J, Hu XQ, Liu N, Wang SX, Zhang Y, Zeng WG, Ni HJ, Zhao B, Chen YF, Tang ZP (2012) Transplantation of adipose-derived stem cells is associated with neural differentiation and functional improvement in a rat model of intracerebral hemorrhage. CNS neuroscience & therapeutics 18 (10):847-854. doi:10.1111/j.1755-5949.2012.00382.x
28. Yang KL, Lee JT, Pang CY, Lee TY, Chen SP, Liew HK, Chen SY, Chen TY, Lin PY (2012) Human adipose-derived stem cells for the treatment of intracerebral hemorrhage in rats via femoral intravenous injection. Cellular & molecular biology letters 17 (3):376-392. doi:10.2478/s11658-012-0016-5

29. Ahn SY, Chang YS, Sung DK, Sung SI, Yoo HS, Lee JH, Oh WI, Park WS (2013) Mesenchymal stem cells prevent hydrocephalus after severe intraventricular hemorrhage. Stroke; a journal of cerebral circulation 44 (2):497-504. doi:10.1161/strokeaha.112.679092
30. Wang SP, Wang ZH, Peng DY, Li SM, Wang H, Wang XH (2012) Therapeutic effect of mesenchymal stem cells in rats with intracerebral hemorrhage: Reduced apoptosis and enhanced neuroprotection. Spandidos Publications Ltd. (10 Vriaxidos Street, Athens 11635, Greece). http://www.spandidos-publications.com/serveFile/mmr_6_4_848_PDF.pdf?type=article&article_id=mmr_6_4_848&item=PDF

http://ovidsp.ovid.com/ovidweb.cgi?T=JS&PAGE=reference&D=emed10&NEWS=N&AN=2012488687. Accessed (Wang, Wang, Peng, Li, Wang, Wang) First Department of Neurology, Dalian Central Hospital, No. 826 Southwest Road, Shahekou District, Dalian, Liaoning 116033, China 6
31. Chen SJ, Tsai JC, Lin TY, Chang CK, Tseng TH, Chien CL (2012) Brain-derived neurotrophic factor-transfected and nontransfected 3T3 fibroblasts enhance migratory neuroblasts and functional restoration in mice with intracerebral hemorrhage. Journal of neuropathology and experimental neurology 71 (12):1123-1136. doi:10.1097/NEN.0b013e3182779e96
32. Seghatoleslam M, Jalali M, Nikravesh MR, Hosseini M, Hamidi Alamdari D, Fazel A (2012) Therapeutic benefit of intravenous administration of human umbilical cord blood- mononuclear cells following intracerebral hemorrhage in rat. Iranian journal of basic medical sciences 15 (3):860-872
33. Vaquero J, Otero L, Bonilla C, Aguayo C, Rico MA, Rodriguez A, Zurita M (2013) Cell therapy with bone marrow stromal cells after intracerebral hemorrhage: impact of platelet-rich plasma scaffolds. Cytotherapy 15 (1):33-43. doi:10.1016/j.jcyt.2012.10.005
34. Qin J, Gong G, Sun S, Qi J, Zhang H, Wang Y, Wang N, Wang QM, Ji Y, Gao Y, Shi C, Yang B, Zhang Y, Song B, Xu Y (2013) Functional recovery after transplantation of induced pluripotent stem cells in a rat hemorrhagic stroke model. Neuroscience letters 554:70-75. doi:10.1016/j.neulet.2013.08.047
35. Qin J, Song B, Zhang H, Wang Y, Wang N, Ji Y, Qi J, Chandra A, Yang B, Zhang Y, Gong G, Xu Y (2013) Transplantation of human neuro-epithelial-like stem cells derived from induced pluripotent stem cells improves neurological function in rats with experimental intracerebral hemorrhage. Neuroscience letters 548:95-100. doi:10.1016/j.neulet.2013.05.007
36. Liang H, Yin Y, Lin T, Guan D, Ma B, Li C, Wang Y, Zhang X (2013) Transplantation of bone marrow stromal cells enhances nerve regeneration of the corticospinal tract and improves recovery of neurological functions in a collagenase-induced rat model of intracerebral hemorrhage. Korean Society for Molecular and Cellular Biology (635-4 Yeogsam-dong,Kangnam-gu, Seoul 135-703, South Korea). http://ovidsp.ovid.com/ovidweb.cgi?T=JS&PAGE=reference&D=emed12&NEWS=N&AN=2014075000. Accessed (Liang, Yin, Lin, Guan, Li, Wang, Zhang) Key Laboratory of Neurosurgery, College of Heilongjiang Province, First Affiliated Hospital of Harbin Medical University, Harbin, China 36
37. Bao XJ, Liu FY, Lu S, Han Q, Feng M, Wei JJ, Li GL, Zhao RC, Wang RZ (2013) Transplantation of Flk-1+ human bone marrow-derived mesenchymal stem cells promotes behavioral recovery and anti-inflammatory and angiogenesis effects in an intracerebral hemorrhage rat model. International journal of molecular medicine 31 (5):1087-1096. doi:10.3892/ijmm.2013.1290
38. Seghatoleslam M, Jalali M, Nikravesh MR, Hamidi Alamdari D, Hosseini M, Fazel A (2013) Intravenous administration of human umbilical cord blood-mononuclear cells dose-dependently relieve neurologic deficits in rat intracerebral hemorrhage model. Annals of anatomy = Anatomischer Anzeiger : official organ of the Anatomische Gesellschaft 195 (1):39-49. doi:10.1016/j.aanat.2012.05.002

39. Wakai T, Sakata H, Narasimhan P, Yoshioka H, Kinouchi H, Chan PH (2014) Transplantation of neural stem cells that overexpress SOD1 enhances amelioration of intracerebral hemorrhage in mice. Journal of cerebral blood flow and metabolism : official journal of the International Society of Cerebral Blood Flow and Metabolism 34 (3):441-449. doi:10.1038/jcbfm.2013.215
40. Lee HS, Kim KS, Lim HS, Choi M, Kim HK, Ahn HY, Shin JC, Joe YA (2015) Priming Wharton's jelly-derived mesenchymal stromal/stem cells with Rock inhibitor improves recovery in an intracerebral hemorrhage model. Wiley-Liss Inc. http://onlinelibrary.wiley.com/journal/10.1002/(ISSN)1097-4644

http://ovidsp.ovid.com/ovidweb.cgi?T=JS&PAGE=reference&D=emed12&NEWS=N&AN=2014966937. Accessed (Lee, Kim, Lim, Choi, Kim, Joe) Cancer Research Institute, Department of Medical Life Sciences, Catholic University of Korea, Seoul 137-701, South Korea 116
